# Pulmonary *Nocardia ignorata* Infection in Gardener, Iran, 2017

**DOI:** 10.3201/eid2603.180725

**Published:** 2020-03

**Authors:** Hossein A. Rahdar, Mehrnaz A. Gharabaghi, Abbas Bahador, Shahram Shahraki-Zahedani, Morteza Karami-Zarandi, Shahram Mahmoudi, Mohammad M. Feizabadi

**Affiliations:** Iranshahr University of Medical Sciences, Iranshahr, Iran (H.A. Rahdar);; Tehran University of Medical Sciences, Tehran, Iran (M.A. Gharabaghi, A. Bahador, M. Karami-Zarandi, S. Mahmoudi, M.M. Feizabadi);; Zahedan University of Medical Sciences, Zahedan, Iran (S. Shahraki-Zahedani)

**Keywords:** Nocardia ignorata, bacteria, Actinomycetales, Nocardiaceae, emerging communicable diseases, infections, pulmonary nocardiosis, soil, gardener, respiratory infections, Iran

## Abstract

*Nocardia ignorata,* which was first described in 2001, is a rare human pathogen. We report a case of pulmonary nocardiosis caused by this bacterium in a 55-year-old man from Iran. The patient, a gardener, had frequent exposure to soil and may have acquired the infection from that source.

Since the description of *Nocardia ignorata* in 2001 (*1*), 4 respiratory isolates in Europe and 3 corneal isolates in India have been reported (*2*,*3*). Among the respiratory isolates, only 2 were confirmed as the cause of disease (*2*). We report a case of pulmonary nocardiosis that was caused by *Nocardia ignorata* in a person from Iran.

The patient, a 55-year-old man, smoked, had multiple myeloma, and had a history of opium use for 3 years. He was consuming methadone syrup daily at the time of hospitalization but had no history of corticosteroid consumption. He had frequent exposure to dust because of working in a garden.

The recent course of disease began ≈4 months earlier, initially as a feeling of heaviness and pain in the anterior chest (no clear distribution) and shortness of breath (functional class II level). However, coronary disease was rejected as a possible diagnosis during examination. The patient had night sweats and a weight loss of 5% in the past 4 months.

Two months after initial symptoms began, he had had fever, chills, and gradually productive coughs. During a visit to a different hospital, he was given a diagnosis of bacterial pneumonia and received azithromycin and cefixime, which initially reduced the symptoms but did not completely resolve them. When he sought care at Imam Khomeini Hospital (Tehran, Iran) because he had symptoms including cramping abdominal pain, fever, and shortness of breath 4–7 days before the visit, he was first admitted to the emergency department and then transferred to the lung unit.

Vital signs of the patient at the time of hospitalization were temperature 37.6°C, blood pressure 135/75 mm Hg, heart rate 80 beats/min, respiratory rate 18 breaths/min, and O_2_ saturation 90%. Laboratory findings were increased leukocyte count (12,210 cells/mm^3^ with 68.8% neutrophils) and C-reactive protein level 65 mg/L (reference value <5 mg/L); thyroid function test results were within reference ranges. Examination of abdominal organs did not show organomegaly or any other indications of disease; prostate size and consistency were unremarkable by testicular and digital rectal examinations. Heart sounds were unremarkable, and cervical, axillary, and extracorporeal lymph nodes were also unremarkable. Examination of the lungs showed fine end inspiratory crackles in both lungs.

Chest radiograph showed a mediastinal mass from the periphery of the right main bronchus to the medial and lower branches and an extension to the posterior part (vertebral trunk and adjacent to the esophagus). The maximum size of the mass was 31 × 74 mm, and an air bronchogram showed opacities near the mass, suggesting postobstructive pneumonia.

The patient was given empirical pneumonia treatment with levofloxacin (750 mg/d) and ceftriaxone (1 g, 2×/d) for 8 days. For the mediastinal mass, the patient underwent endoscopic ultrasonography and bronchoscopy, but his symptoms did not resolve. In a spiral computed tomography scan of the thorax, we found patchy ground glass and nodular opacities that were spread in both lungs, especially in posterior and lateral segments of the left lower lobe, the posterior segment of the right lower lobe, lingular, and the right upper lobe. Thus, nocardiosis and tuberculosis examinations were recommended.

We performed a tuberculin skin test, and the intraderm response to tuberculin was absent. Moreover, mucosal erythema with inflammation were seen on a right lung bronchoscopic examination. We found abundant mucoid secretions, no endobronchial ulcers, and a white discharge without endobronchial ulcer in the left lung.

We then subjected a bronchoalveolar lavage specimen for microbiological cultures. No *Mycobacterium* growth was seen, but colonies suspected of being *Nocardia* species appeared on blood agar plates after 4 days of incubation. A modified Kinyoun acid-fast stain confirmed the partial acid-fast staining feature of the colonies.

We performed DNA extraction by using the modified Pitcher method (*4**–**6*) and a *Nocardia*-specific PCR with primers NG1 (5′-ACCGACCACAAGGGGG-3′) and NG2 (5′-GGTTGTAAACCTCTTTCGA-3′). We obtained positive results with this PCR and a resistance-to-lysozyme test, and confirmed the presence of *Nocardia* species. For accurate identification, we PCR amplified 16S rRNA and partial DNA gyrase B (*gyrB*) genes (*4*) and subjected PCR products to direct sequencing (GenBank accession nos. KY817987.1 for 16S rRNA and MN159177 for *gyrB*).

We performed a BLAST analysis (https://blast.ncbi.nlm.nih.gov/Blast.cgi) by using the megablast algorithm. This analysis confirmed the identity as *N. ignorata* (16S rRNA coverage 100% and 99% identity with GenBank accession no. KM113026.1; *gyrB* coverage 100% and 100% identity with GenBank accession no. GQ496109.1).

Antimicrobial drug susceptibility testing (broth microdilution method) showed that the isolate was susceptible to trimethoprim/sulfamethoxazole, imipenem, amikacin, doxycycline, and linezolid and resistant to erythromycin, ceftriaxone, and ciprofloxacin. At this point, we gave the patient cotrimoxazole and imipenem for 20 days and then trimethoprim/sulfamethoxazole for 6 months. The clinical, biological, and radiologic outcomes of the treatment were optimal, and no recurrence occurred after 8 months.

*N. ignorata* is a rare human pathogen. However, soil samples have been described as a possible reservoir (*2*,*7*,*8*). Accordingly, this patient, who had frequent exposure to soil through his work as a gardener, might have acquired the infection from that source.
